# Role of leukocyte parameters in patients with ST-segment elevation myocardial infarction undergoing primary percutaneous coronary intervention with high thrombus burden

**DOI:** 10.3389/fcvm.2024.1397701

**Published:** 2024-06-18

**Authors:** Hao Wang, Shixing Li, Jin Yu, Jingsong Xu, Yan Xu

**Affiliations:** Department of Cardiology, Second Affiliated Hospital of Nanchang University, Nanchang, Jiangxi Province, China

**Keywords:** leukocyte parameters, ST-segment elevation myocardial infarction, high thrombus burden, NLR, MLR

## Abstract

**Objective:**

Leukocyte parameters are associated with cardiovascular diseases. The aim of the present study was to investigate the role of leukocyte parameters in patients with ST-segment elevation myocardial infarction (STEMI) undergoing primary percutaneous coronary intervention (PPCI) with high thrombus burden (HTB).

**Methods:**

A total of 102 consecutive STEMI patients with HTB who underwent PPCI within 12 h from the onset of symptoms between June 2020 and September 2021 were enrolled in this study. In addition, 101 age- and sex-matched STEMI patients with low thrombus burden (LTB) who underwent PPCI within 12 h from the onset of symptoms were enrolled as controls. Leukocyte parameters, such as neutrophil to lymphocyte ratio (NLR), platelet to lymphocyte ratio (PLR), and monocyte to lymphocyte ratio (MLR), were calculated at the time of admission.

**Results:**

The value of NLR and MLR were significantly higher in the HTB group than in the LTB group (6.24 ± 4.87 vs. 4.65 ± 3.47, *p* = 0.008; 0.40 ± 0.27 vs. 0.33 ± 0.20, *p* = 0.038). A cutoff value of >5.38 for NLR had a sensitivity and specificity of 53.9% and 74.3%, respectively, and MLR >0.29 had a sensitivity and specificity of 60.8% and 55.4%, respectively, for determining the STEMI patients with HTB [area under the receiver operating characteristic curve (AUC): 0.603, 95% confidence interval (CI): 0.524–0.681, *p* = 0.012; AUC: 0.578, 95% CI: 0.499–0.656, *p* = 0.046]. There was no significant difference of all-cause mortality rate and major adverse cardiac events (MACEs) between the STEMI patients with HTB or with LTB (3.92% in HTB group vs. 2.97% in LTB group, *p* = 0.712; 10.78% in HTB group vs. 8.91% in LTB group, *p* = 0.215). Compared with the HTB patients in the low NLR group, C-reactive protein, baseline troponin I, baseline brain natriuretic peptide, and leukocyte parameters, such as white blood cell, neutrophil, lymphocyte, NLR, PLR, and MLR, were also significantly higher in the high NLR group in STEMI patients who underwent PPCI with HTB (18.94 ± 19.06 vs. 35.23 ± 52.83, *p* = 0.037; 10.99 ± 18.07 vs. 21.37 ± 19.64, *p* = 0.007; 199.39 ± 323.67 vs. 430.72 ± 683.59, *p* = 0.028; 11.55 ± 3.56 vs. 9.31 ± 2.54, *p* = 0.001; 9.77 ± 3.17 vs. 5.79 ± 1.97, *p* = 0.000; 1.16 ± 0.44 vs. 2.69 ± 1.23, *p* = 0.000; 9.37 ± 4.60 vs 1.31 ± 2.58, *p* = 0.000; 200.88 ± 89.90 vs. 97.47 ± 50.99, *p* = 0.000; 0.52 ± 0.29 vs. 0.26 ± 0.14, *p* = 0.000, respectively). MACEs and heart failure in the high NLR group were significantly higher than that in the low NLR group of STEMI patients who underwent PPCI with HTB (20.45% vs. 4.25%, *p* = 0.041; 10.91% vs. 2.13%, *p* = 0.038).

**Conclusion:**

The value of NLR and MLR were higher in STEMI patients who underwent PPCI with HTB. In STEMI patients who underwent PPCI with HTB, a raised NLR could effectively predict the occurrence of MACEs and heart failure.

## Introduction

ST-segment elevation myocardial infarction (STEMI) is one of the most serious cardiovascular diseases and has the highest mortality rate. Primary percutaneous coronary intervention (PPCI) could rapidly open the infarct-related artery and is recognized as the most effective strategy for the treatment of STEMI (1). However, high thrombus burden (HTB) may lead to more complications during the procedure, such as no-reflow, slow-reflow, or stent thrombosis, which could negatively impact long-term survival (2–4). Therefore, it is important to identify related biomarkers to distinguish high thrombotic status in patients with STEMI.

Inflammation is believed to contribute to thrombus formation and progression and is one of the cornerstones of STEMI pathophysiology (5). Leukocyte parameters, such as leukocyte count, neutrophil count, neutrophil to lymphocyte ratio (NLR), platelet to lymphocyte ratio (PLR), and monocyte to lymphocyte ratio (MLR), reflect the inflammatory status and are appreciated as inflammation markers that have been associated with acute coronary syndrome (6, 7). NLR and MLR were found to have a slight positive correlation with high-risk STEMI mortality (8). The value of NLR and PLR on admission could predict the in-hospital major adverse cardiac events (MACEs) in patients with acute myocardial infarction (9). The leukocyte count, neutrophil count, and NLR were higher in acute myocardial infarction patients with thrombus burden than those without thrombus burden; neutrophil count was independently associated with thrombus burden (10). PLR had also been shown to be associated with no-reflow in STEMI patients (11). However, there is a lack of data regarding the role of leukocyte parameters in STEMI patients with HTB. Leukocyte parameters can be obtained by routine blood tests, which are among the preferred laboratory tests for STEMI patients, and the results can be acquired rapidly, in as little as 10 min. We sought to investigate the predictive value of leukocyte parameters in STEMI patients who underwent PPCI with HTB.

## Materials and methods

### Ethics statement

The present study was approved by the Ethics Committee of the Second Affiliated Hospital of Nanchang University (approval no. 2022-07). The investigation complied with the principles outlined in the Declaration of Helsinki and its later amendments or comparable ethical standards.

### Study population

This was a single-center, retrospective study conducted at the Second Affiliated Hospital of Nanchang University in China. A total of 102 consecutive STEMI patients with HTB who underwent PPCI within 12 h from the onset of symptoms between June 2020 and September 2021 were enrolled in this study. In addition, 101 age- and sex-matched STEMI patients who underwent PPCI with low thrombus burden (LTB) within 12 h from the onset of symptoms were enrolled as controls. STEMI was defined as >30 min of persistent chest pain and ST-segment elevation of >2 mm in at least two contiguous leads or the presence of a new bundle branch block. The state of the HTB group was classified as greatest dimension of thrombus >2 vessel diameters or total vessel occlusion due to thrombus. The LTB group was categorized as having no thrombus, or potential thrombus, or the greatest dimension of thrombus <0.5 vessel diameter, or the greatest dimension >0.5 to <2 vessel diameters (12).

### Study intervention

A loading dose of aspirin and P2Y12 inhibitor (ticagrelor or clopidogrel) were given before the intervention. All patients had undergone PPCI in accordance with standard guideline recommendations. Thrombus aspiration (TA) was performed in STEMI patients with HTB. A Thrombuster II aspiration catheter was used to aspirate the thrombus (pull and push). An aspiration catheter was reintroduced into the infarct-related artery beyond the thrombus. All patients received 0.5 mg tirofiban and 0.2 mg nitroglycerin intracoronary injection after restoring blood flow to the infarct-related artery. Tirofiban and nitrate were not administered in STEMI patients after PPCI. MACEs were defined as all-cause mortality, cardiovascular death, reinfarction, heart failure, or target vessel revascularization. The occurrence of MACEs within 1 year was assessed using medical electronic records. If no follow-up data were available, telephonic follow-up was used to determine the occurrence of MACEs within 1 year.

### Laboratory analysis

Samples of peripheral venous blood were drawn for complete blood count, lipid, and biochemistry before coronary angiography. The NLR, PLR, and MLR were calculated as the preprocedural ratio of neutrophil, platelet, and monocyte to lymphocyte, which were obtained from the same blood sample. Baseline clinical characteristics, laboratory data, lesion and procedural characteristics, and the occurrence of MACEs obtained using patient records and registries.

### Statistical analysis

The Kolmogorov–Smirnov test was used to assess the normality of continuous variables. Continuous variables were summarized as mean ± standard deviation (SD) and were analyzed parametrically using independent-sample Student’s *t*-tests or non-parametrically using Mann–Whitney U-tests. Categorical variables were expressed as frequencies and percentages and were compared using Pearson’s chi-square test or Fisher's exact test. Receiver operating characteristic (ROC) curves were performed to find the optimal cutoff values for the role of NLR and MLR in STEMI patients who underwent PPCI with HTB. All statistical tests were two-tailed and a p-value < 0.05 was considered statistically significant. Statistical analyses were performed using SPSS 26.0 (IBM Corp., New York, NY, USA).

## Results

Among 506 patients with STEMI, 102 STEMI patients with HTB who underwent PPCI within 12 h from the onset of symptoms were included in the present study as well as 101 age- and sex-matched STEMI patients with LTB who underwent PPCI within 12 h from the onset of symptoms as controls. The baseline clinical and hemodynamic characteristics of the study population are shown in Table 1. Of the 203 participants, 101 (49.8%) had hypertension and 60 (29.6%) had diabetes mellitus. The mean age of the participants was 60.24 years; most patients (86.7%) were men. No significant differences were observed between the HTB and LTB groups in terms of medical history, baseline medication, serum concentrations [creatinine, H1bc, C-reactive protein (CRP), troponin I, brain natriuretic peptide (BNP), and lipids], and baseline left ventricular ejection fraction (LVEF) (p > 0.05). Compared with STEMI patients with LTB, the percentage of thrombolysis in myocardial infarction (TIMI) flow grade 3 immediately after PPCI was higher than those with HTB (98.02% vs. 92.08%, *p* = 0.05). There was no significant difference in other lesions and procedural characteristics between the HTB group and the LTB group in STEMI patients who underwent PPCI (p > 0.05) (Table 2). The value of NLR and MLR were significantly higher in the HTB group than in the LTB group (6.24 ± 4.87 vs. 4.65 ± 3.47, *p* = 0.008; 0.40 ± 0.27 vs. 0.33 ± 0.20, *p* = 0.038). Furthermore, there were no significant differences between the groups in terms of white blood cell (WBC), neutrophil, lymphocyte, platelet, monocyte, and PLR (p > 0.05) (Table 3).

**Table 1:** Baseline clinical and hemodynamic characteristics of the study population before PPCI.

	HTB (*N* = 102)	LTB (*N* = 101)	p-value
Men, *n* (%)	90 (88.24%)	86 (85.15%)	0.520
Age	61.36 ± 14.12	59.10 ± 12.72	0.232
BMI	22.69 ± 2.91	22.97 ± 2.64	0.475
Medical history			
Diabetes mellitus, *n* (%)	31 (30.39%)	29 (28.71%)	0.794
Hypertension, *n* (%)	48 (47.83%)	53 (47.22%)	0.443
Hypercholesterolemia, *n* (%)	31 (30.39%)	36 (35.64%)	0.429
Current smoker, *n* (%)	50 (49.02%)	43 (42.57%)	0.359
Family history of CAD, *n* (%)	2 (1.96%)	3 (2.97%)	0.645
Previous myocardial infarction, *n* (%)	1 (0.98%)	2 (1.98%)	0.557
Previous CABG, *n* (%)	0	0	
Previous PCI, *n* (%)	3 (2.94%)	6 (5.94%)	0.302
Previous stroke, *n* (%)	10 (9.80%)	7 (6.93%)	0.462
Baseline medication			
Anti-platelet drugs, *n* (%)	99 (97.06%)	99 (98.02%)	0.661
Statins, *n* (%)	98 (96.08%)	99 (98.02%)	0.417
ACEI/ARB/ARNI, *n* (%)	55 (53.92%)	66 (65.35%)	0.098
Serum concentrations			
Creatinine, mmol/L	92.97 ± 65.84	83.64 ± 25.13	0.185
eGFR	90.37 ± 31.91	90.67 ± 24.70	0.940
H1bc, %	6.55 ± 1.70	6.49 ± 1.55	0.782
CRP	27.72 ± 41.53	24.59 ± 37.08	0.572
Troponin I, ng/ml	16.59 ± 19.54	14.63 ± 19.55	0.476
BNP, pg/ml	324.13 ± 557.66	365.26 ± 1,004.88	0.718
TC, mmol/L	4.62 ± 1.19	4.79 ± 1.20	0.341
TG, mmol/L	1.85 ± 1.52	2.20 ± 1.99	0.160
LDL-C, mmol/L	2.81 ± 0.95	2.97 ± 1.02	0.243
Baseline LVEF, (%)	52.00 ± 7.59	52.88 ± 8.36	0.095

CAD, coronary artery disease; CABG, coronary artery bypass grafting; ACEI, angiotensin converting enzyme inhibitors; ARB, angiotensin receptor blocker; ARNI, angiotensin receptor neprilysin inhibitor.

**Table 2:** Lesion and procedural characteristics of the study population.

** **	HTB (*N* = 102)	LTB (*N* = 101)	p-value
Vessel treated			
MVD, *n* (%)	39 (38.24%)	49 (48.51%)	0.184
SVD, *n* (%)	32 (31.37%)	28 (27.72%)	0.518
Vessel name			
LM, *n* (%)	2 (1.96%)	1 (1.00%)	0.569
LAD, *n* (%)	51 (50.00%)	62 (61.39%)	0.141
LCX, *n* (%)	11 (10.78%)	12 (11.88%)	0.806
RA, *n* (%)	43 (42.16%)	34 (33.66%)	0.217
Lesion length, mm	31.03 ± 14.37	35.06 ± 19.37	0.094
Stent length, mm	36.38 ± 15.69	39.69 ± 18.73	0.172
Stent number	1.39 ± 0.75	1.54 ± 0.84	0.174
No-reflow*, n (%)*	1 (0.99%)	6 (5.94%)	0.053
TA, *n* (%)	102 (100%)	0 (0%)	
TIMI flow post PCI immediately			
0 or I, *n* (%)	0	1 (0.99%)	0.320
II, *n* (%)	2 (1.98%)	8 (2.97%)	0.05*
III, *n* (%)	101 (98.02%)	93 (92.08%)	0.05*
No-reflow or slow reflow, *n* (%)	1	7	
TIMI flow post PCI at last			
0 or I, *n* (%)	0	0	
II, *n* (%)	1 (0.99%)	3 (2.97%)	0.312
III, *n* (%)	101 (99.01%)	98(97.03%)	0.312

SVD, single vessel disease; LM, left main artery; LAD, left anterior descending artery; LCX, left circumflex artery; RCA, right coronary artery.

**Table 3:** Leukocyte parameters of the study population.

	HTB (*N* = 102)	LTB (*N* = 101)	p-value
White blood cell	10.51 ± 3.32	10.22 ± 3.17	0.523
Neutrophil	7.93 ± 3.33	7.26 ± 2.85	0.125
Lymphocyte	1.87 ± 0.44	2.21 ± 1.42	0.062
Platelet	213.45 ± 59.27	220.65 ± 57.11	0.379
Monocyte	0.59 ± 0.29	0.59 ± 0.26	0.966
NLR	6.24 ± 4.87	4.65 ± 3.47	0.008**
PLR	153.23 ± 90.49	131.84 ± 75.31	0.069
MLR	0.40 ± 0.27	0.33 ± 0.20	0.038*

* Indicates significance at the 5% level.

** Indicates significance at the 1% level.

**Figure 1:**
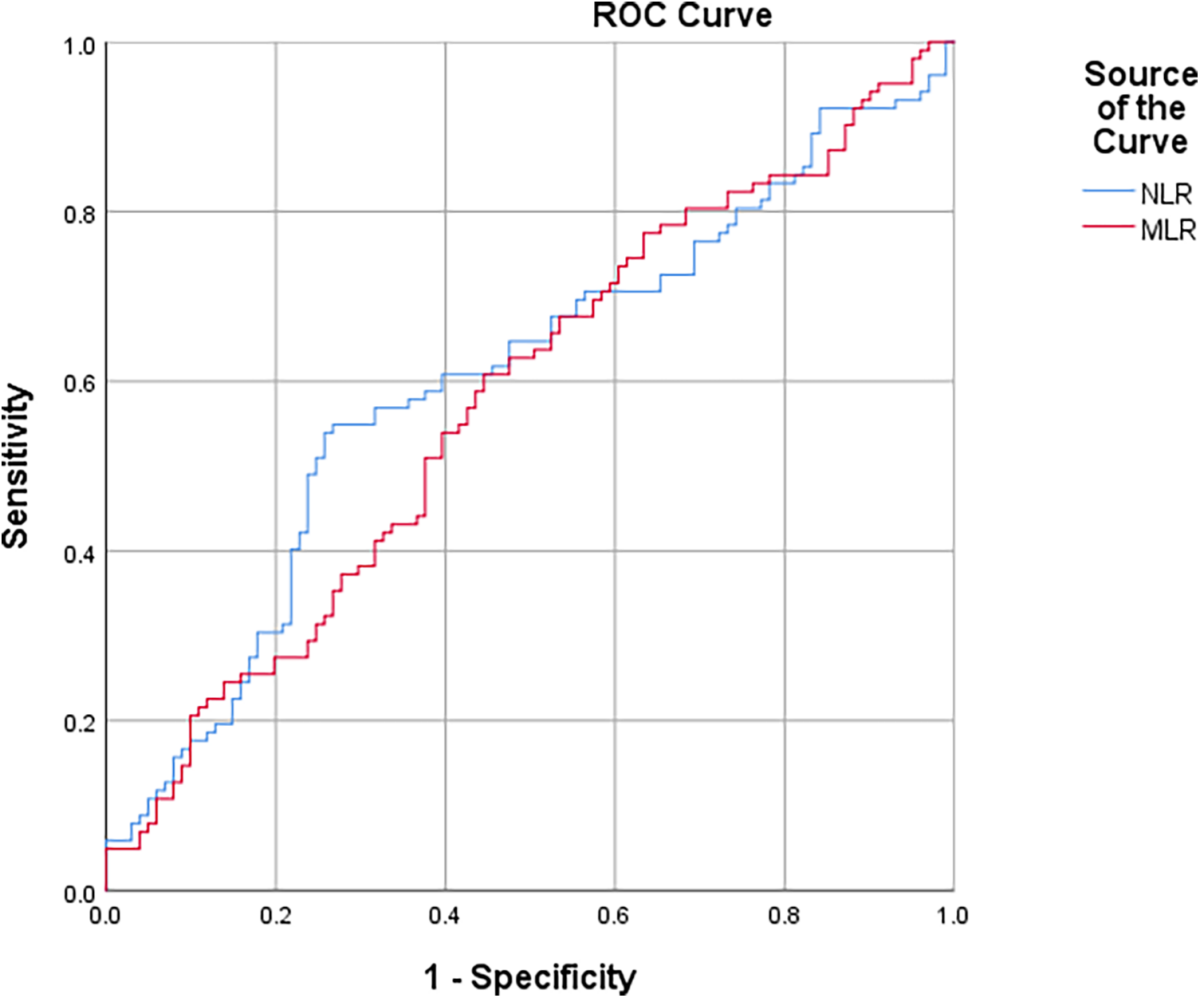
ROC curve of NLR and MLR for predicting STEMI patients undergoing primary PCI with high thrombus burden.

In the ROC curve analysis, a cutoff value of >5.38 for NLR had a sensitivity and specificity of 53.9% and 74.3%, respectively, and MLR >0.29 had a sensitivity and specificity of 60.8% and 55.4%, respectively, for determining the STEMI patients with HTB [area under the ROC curve (AUC): 0.603, 95% CI: 0.524–0.681, *p* = 0.012; AUC: 0.578, 95% CI: 0.499–0.656, *p* = 0.046] (Figure 1).

During the median follow-up of 365 days, 7 deaths and 20 MACEs were reported. There was no significant difference in all-cause mortality rate and MACEs between the STEMI patients with HTB or those with LTB (3.92% in the HTB group vs. 2.97% in the LTB group, *p* = 0.712; 10.78% in the HTB group vs. 8.91% in the LTB group, *p* = 0.215).

A subgroup analysis was used to investigate the role of NLR in STEMI patients with HTB. Patients were stratified into the high or low NLR groups based on the cutoff value. There were 47 STEMI patients with HTB in the low NLR group and 55 in the high NLR group. No significant differences were found between the low and high NLR groups regarding age, sex, body mass index (BMI), medical history, baseline medication, multi-vessel disease (MVD), creatinine, estimated glomerular filtration rate (eGFR), H1bc, total cholesterol (TC), triglyceride (TG), low-density lipoprotein (LDL-C), platelet, monocyte, and baseline LVEF (Supplementary Table S1). The levels of CRP (18.94 ± 19.06 vs. 35.23 ± 52.83, p = 0.037), baseline troponin I (10.99 ± 18.07 vs. 21.37 ± 19.64, p = 0.007), and baseline BNP (199.39 ± 323.67 vs. 430.72 ± 683.59, *p* = 0.028) were significantly higher in the high NLR group. Compared with the patients in the low NLR group, leukocyte parameters, such as WBC, neutrophil, lymphocyte, NLR, PLR, and MLR, were also significantly higher in the high NLR group (11.55 ± 3.56 vs. 9.31 ± 2.54, *p* = 0.001; 9.77 ± 3.17 vs. 5.79 ± 1.97, p = 0.000; 1.16 ± 0.44 vs. 2.69 ± 1.23, *p* = 0.000; 9.37 ± 4.60 vs 1.31 ± 2.58, *p* = 0.000; 200.88 ± 89.90 vs. 97.47 ± 50.99, *p* = 0.000; 0.52 ± 0.29 vs. 0.26 ± 0.14, *p* = 0.000) (Table 4). During the follow-up period, nine (20.45%) patients had MACEs and six (10.91%) had heart failure in the high NLR group, and two (4.25%) had MACEs and one (2.13%) had heart failure in the low NLR group. The occurrence of MACEs and heart failure in the high NLR group were significantly higher than that in the low NLR group in STEMI patients with HTB (*p* = 0.041 and *p* = 0.038) (Table 5). Furthermore, three (2.94%) patients had all-cause mortality, one (0.98%) had cardiovascular death, one (0.98%) had target vessel revascularization, and one (0.98%) had reinfarction; no statistical differences were observed between the groups (5.45% vs. 2.13%, *p* = 0.393; 1.82% vs. 0%, *p* = 0.322; 1.82% vs. 0%, *p* = 0.322; 1.82% vs. 0%, *p* = 0.322).

**Table 4:** Cutoff and AUC value as a result of ROC analysis.

Parameter	Sensitivity (%)	Specificity (%)	Area under curve	Cutoff value
NLR	53.9%	74.3%	0.603	5.38
MLR	60.8%	55.4%	0.578	0.29

**Table 5:** One-year clinical outcomes of high thrombus burden patients with low and high NLR.

** **	Low NLR (≤5.38) (*N* = 47)	High NLR (>5.38) (*N* = 55)	p-value
All-cause mortality, *n* (%)	1 (2.13%)	2 (3.64%)	0.650
Cardiovascular death, *n* (%)	0	1 (1.82%)	0.322
Reinfarction, *n* (%)	0	1 (1.82%)	0.322
Heart failure, *n* (%)	1 (2.13%)	6 (10.91%)	0.038*
Target vessel revascularization, *n* (%)	0	1 (1.82%)	0.322
MACEs, *n* (%)	2 (4.25%)	9 (20.45%)	0.041*

* Indicates significance at the 5% level.

## Discussion

Accumulated evidence has shown that leukocyte parameters such as NLR, PLR, and MLR are simple and reliable markers for diagnosing and predicting the prognosis of cardiovascular diseases (13, 14). This study investigated the predictive value of leukocyte parameters in STEMI patients who underwent PPCI with HTB. The striking findings of the present study were as follows: (1) the value of NLR and MLR were significantly higher in STEMI patients who underwent PPCI with HTB; (2) more participants in the HTB group had a TIMI blood flow of 3 immediately after PPCI compared to those in the LTB group; (3) the level of CRP, baseline troponin I, and baseline BNP were higher in the high NLR group than in the low NLR group in STEMI patients with HTB; and (4) an elevated NLR was associated with MACEs and heart failure in STEMI patients who underwent PPCI with HTB.

Balloon dilatation and stent expansion may result in thrombus defluxion and microthrombus formation, which may lead to no-reflow or slow flow. TA is a simple, adjunctive tool to PPCI and is thought to improve coronary blood flow in STEMI patients with HTB (15). The TAPAS study reported that TA significantly improves myocardial perfusion and outcomes in STEMI patients with HTB (16). However, the TASTE trial and TOTAL trial revealed negative results (17, 18). In our study, we found that the proportion of TIMI grade 2 or grade 3 blood flow immediately after PPCI was higher in STEMI patients who underwent TA with HTB than those who underwent PPCI with LTB. There was no significant difference in all-cause mortality rate and MACEs between the STEMI patients who underwent PPCI with HTB or with LTB. TA was performed on all patients in the HTB group in our study, which reduced the thrombus burden and produced a similar prognosis in patients with LTB. The use of glycoprotein IIb/IIIa inhibitors, such as tirofiban, may have been responsible for the similar TIMI grade 2 or grade 3 blood flow after PPCI at last in the two groups.

It is known that inflammation contributes to coronary thrombus formation and growth, and also augments the consequences of thrombus accumulation, potentially influencing prognosis (19). The leukocyte parameters, which were simple inflammatory markers, could act as a bridge to mitigate the gap in assessing the risk and outcomes in cardiovascular disease (20–22). Leukocyte parameters, such as NLR, neutrophil count, and lymphocyte count, were the independent predictors of coronary thrombus formation in patients with non-ST-segment elevated acute coronary syndrome (23). Similarly, Dolu et al. (24) showed that NLR and PLR were independent predictors for thrombus burden in STEMI patients. In line with the results of these studies, we detected in our study that NLR and MLR were especially higher in STEMI patients who underwent PPCI with HTB. Furthermore, we revealed that an elevated NLR was associated with MACEs and heart failure in STEMI patients who underwent PPCI with HTB. Our study revealed no significant distinction between WBC, neutrophils, lymphocytes, and monocytes between those with HTB and those with LTB. A high neutrophil count reflects inflammation while a low lymphocyte count is associated with stress response. NLR, which combines neutrophils and lymphocytes, reflects the balance between inflammatory activation and immune stress response. NLR, PLR, and MLR were superior to individual indicators for the prediction of cardiovascular disease. PLR also had negative results in our study, partly because of the small sample size and the widened gap between maximum and minimum values.

We found that the baseline LVEF in the HTB and LTB groups was 52% and 52.88%, respectively; there was no significant difference between the baseline LVEF in the two groups. However, Dolu et al. (24) found that low LVEF was associated with HTB. The level of troponin I at admission was 16.59 ng/ml in the HTB group and 14.63 ng/ml in the LTB group. However, the level of troponin I at admission was 21.37 ng/ml in the high NLR group in STEMI patients with HTB and 10.99 ng/ml in the low NLR group in STEMI patients with HTB. The baseline levels of BNP and troponin I showed no significant differences when grouped in HTB or LTB. In particular, the baseline BNP and troponin I were higher in the high NLR group in STEMI patients with HTB than in the low NLR group. Pawłowski et al. also found that the initial level of troponin I could predict thrombus burden in patients with acute coronary syndrome (25). Peak troponin I levels did not produce a positive result in HTB patients with a high NLR. A possible reason was that the upper limit of troponin I in our hospital was 50 ng/ml.

CRP has been suggested as an inflammatory biomarker and is the most commonly used in clinical practice. The CRP level was normal in approximately 40% of STEMI patients, which indicated that the predictive value of CRP for detecting inflammatory status was limited in the overall number of STEMI patients (26). Kaplangoray et al. (27) found that the CRP level was higher in STEMI patients with HTB than in those with LTB. In our study, the comparison of CRP levels between the HTB and LTB groups ended in a negative result. However, we found that the CRP level was significantly higher in the high NLR group of STEMI patients with HTB than in the low NLR group. NLR appears superior to CRP in STEMI patients with HTB. NLR is a better predictor of MACE than CRP in patients with acute coronary syndrome (28, 29). The association between CRP and NLR remains inconclusive. Adatia et al. (30) found that CRP, but not NLR, was predictive of MACEs in STEMI patients. The combination of CRP and NLR to predict MACE in patients with acute coronary syndrome was demonstrated to be better than one alone (29, 31).

Complete revascularization with multi-vessel PCI demonstrated benefit in patients with acute coronary syndrome (32, 33). Nevertheless, we only performed PCI on culprit lesions. The proportion of multi-vessel disease and culprit lesions were similar between the HTB group and the LTB group. Hence, this issue was not expected to influence the analysis and the results.

It is reported that interleukin-6 inhibition ziltivekimab, interleukin-1β inhibition canakinumab, and interleukin-17A inhibition secukinumab were associated with a significant reduction in NLR (34–36). The ongoing ZEUS cardiovascular outcomes trial (Effects of Ziltivekimab vs. Placebo on Cardiovascular Outcomes in Participants With Established Atherosclerotic Cardiovascular Disease, Chronic Kidney Disease, and Systemic Inflammation) will assess whether ziltivekimab can reduce MACEs (37). In the future, research studying the relationship between reducing NLR and reducing cardiovascular events should be considered.

The present study has some limitations. First, our study was retrospective and non-randomized in design; randomized controlled trials are necessary to confirm the results. Second, our study was conducted in a single center with a relatively small number of participants; multi-institutional studies with large samples are needed to validate the findings. Third, leukocyte parameters were measured only on admission in our study; monitoring their dynamic changes may be useful for the indication of disease duration. Finally, intravascular imaging, such as optical coherence therapy or intravascular ultrasound, was not used in our study.

## Conclusion

In conclusion, the NLR and MLR values were higher in STEMI patients who underwent PPCI with HTB. In STEMI patients who underwent PPCI with HTB, a raised NLR could effectively predict the occurrence of MACEs and heart failure.

## Data Availability

The original contributions presented in the study are included in the article/**Supplementary Material**, further inquiries can be directed to the corresponding author.
