# Supplementary material for: Role of leukocyte parameters in patients with ST-segment elevation myocardial infarction undergoing primary percutaneous coronary intervention with high thrombus burden
Source: Front Cardiovasc Med. 2024 Jun 18;11:1397701. doi: 10.3389/fcvm.2024.1397701 (PMC11221325; doi:10.3389/fcvm.2024.1397701)
Supplement: Supplementary file 1 [file Datasheet1.docx]

Supplement Table 1 The baseline characteristics of high thrombus burden patients with low and high NLR

|  | Low NLR (≤5.38)  (N=47) | High NLR (>5.38)  (N=55) | p value |
| --- | --- | --- | --- |
| Men, n(%) | 42(89.36%) | 48(87.27%) | 0.746 |
| Age | 59.51±14.98 | 62.95±13.26 | 0.222 |
| BMI  Medical history | 22.80±2.89 | 22.59±2.96 | 0.722 |
| Diabetes mellitus, n(%) | 13(27.66%) | 18(32.73%) | 0.582 |
| Hypertension, n(%) | 22(46.81%) | 26(47.27%) | 0.963 |
| Hypercholesterolemia, n(%) | 20(42.55%) | 18(32.73%) | 0.213 |
| Current smoker, n(%) | 26(55.32%) | 24(43.64%) | 0.244 |
| Family history of CAD, n(%) | 2(4.26%) | 0 | 0.125 |
| Previous myocardial infarction, n(%) | 0 | 1(1.82%) | 0.322 |
| Previous CABG, n(%) | 0 | 0 |  |
| Previous PCI, n(%) | 0 | 3(5.45%) | 0.106 |
| Previous Stroke, n(%) | 5(10.64%) | 5(9.09%) | 0.796 |
| Baseline medication |  |  |  |
| Anti-platelet drugs, n(%) | 45(95.74%) | 54(98.18%) | 0.473 |
| Statins, n(%) | 45(95.74%) | 53(96.36%) | 0.874 |
| ACEI/ARB/ARNI, n(%) | 28(59.57%) | 27(49.09%) | 0.294 |
| MVD, n(%) | 14(29.79%) | 16(29.09%) | 0.939 |
| Creatinine, mmol/L | 101.89±89.71 | 85.35±33.53 | 0.207 |
| eGFR | 86.56±29.00 | 93.63±34.12 | 0.267 |
| H1bc, % | 6.43 ± 1.61 | 6.66 ± 1.78 | 0.498 |
| TC, mmol/L | 4.71±1.28 | 4.55±1.17 | 0.514 |
| TG, mmol/L | 1.97±1.34 | 1.75±1.66 | 0.469 |
| LDL-c, mmol/L | 2.87±0.94 | 2.75±0.97 | 0.559 |
| Troponin I, ng/ml | 10.99± 18.07 | 21.37 ± 19.64 | 0.007** |
| Troponin I peak, ng/ml | 38.11± 17.51 | 42.40 ± 13.17 | 0.162 |
| BNP, pg/ml | 199.39 ± 323.67 | 430.72 ±683.59 | 0.028* |
| CRP | 18.94±19.06 | 35.23±52.83 | 0.037* |
| White blood cell | 9.31±2.54 | 11.55±3.56 | 0.001** |
| Neutrophil | 5.79±1.97 | 9.77±3.17 | 0.000** |
| Lymphocyte | 2.69±1.23 | 1.16±0.44 | 0.000** |
| Platelet | 220.21±56.58 | 207.67±61.39 | 0.286 |
| Monocyte | 0.64±0.28 | 0.57±0.29 | 0.161 |
| NLR | 1.31 ± 2.58 | 9.37 ± 4.60 | 0.000** |
| PLR | 97.47± 50.99 | 200.88 ± 89.90 | 0.000** |
| MLR | 0.26 ± 0.14 | 0.52± 0.29 | 0.000** |
| Baseline LVEF, (%) | 53.21±7.30 | 50.96±7.74 | 0.135 |

Abbreviations: NLR, neutrophil to lymphocyte ratio; BMI, Body Mass Index; CAD, coronary artery disease; CABG, Coronary Artery Bypass Grafting; PCI, percutaneous coronary intervention; ACEI, angiotensin converting enzyme inhibitors; ARB, Angiotensin Receptor Blocker; ARNI, Angiotensin Receptor Neprilysin Inhibitor; MVD, multi-vessel disease; eGFR, estimated glomerular filtration rate; TC, total cholesterol; TG, Triglyceride; LDL-c, Low-density lipoprotein; BNP, Brain natriuretic peptide; CRP, C-reactive protein; PLR, platelet to lymphocyte ratio; MLR, Monocyte to lymphocyte ratio；LVEF, left ventricular ejection fraction;
